# Electric-Circuit Realization of Fast Quantum Search

**DOI:** 10.34133/2021/9793071

**Published:** 2021-07-26

**Authors:** Naiqiao Pan, Tian Chen, Houjun Sun, Xiangdong Zhang

**Affiliations:** ^1^Key Laboratory of Advanced Optoelectronic Quantum Architecture and Measurements of Ministry of Education, Beijing Key Laboratory of Nanophotonics & Ultrafine Optoelectronic Systems, School of Physics, Beijing Institute of Technology, Beijing 100081, China; ^2^Beijing Key Laboratory of Millimeter Wave and Terahertz Techniques, School of Information and Electronics, Beijing Institute of Technology, Beijing 100081, China

## Abstract

Quantum search algorithm, which can search an unsorted database quadratically faster than any known classical algorithms, has become one of the most impressive showcases of quantum computation. It has been implemented using various quantum schemes. Here, we demonstrate both theoretically and experimentally that such a fast search algorithm can also be realized using classical electric circuits. The classical circuit networks to perform such a fast search have been designed. It has been shown that the evolution of electric signals in the circuit networks is analogies of quantum particles randomly walking on graphs described by quantum theory. The searching efficiencies in our designed classical circuits are the same to the quantum schemes. Because classical circuit networks possess good scalability and stability, the present scheme is expected to avoid some problems faced by the quantum schemes. Thus, our findings are advantageous for information processing in the era of big data.

## 1. Introduction

Quantum computation has been the focus of numerous studies and is expected to play an important role in future information processing, since it outperforms classical computation at many tasks. One such task is quantum searching, introduced by Grover [1]. Given an oracle function *f*(*x*): {1, ⋯, *N*}⟶{0, 1} satisfying *f*(*x*) = 1 if and only if *x* = *w*, Grover's search algorithm can find the value of *w* using of order $\sqrt{N}$ queries. It shows quadratic speedup to the fastest classical algorithm in searching unsorted databases. Such an algorithm is extremely important, both from fundamental and practical standpoints, because it is a basis of many other quantum algorithms [2].

**Figure 1:**
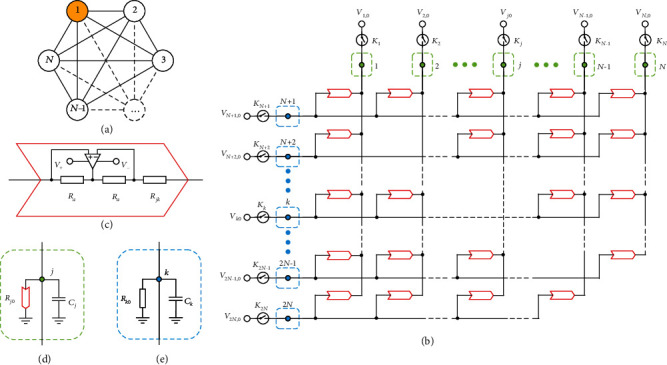
Electrical circuit exhibiting quantum search on a complete graph with nodes of the circuit indicated by green and blue dots. (a) The structure of a complete graph with *N* vertices. Each vertex is connected to all other vertices. The target vertex is labeled by orange. (b) The electrical circuit designed for the quantum search on the complete graph with *N* vertices. The 2*N* nodes are labeled in order and are connected to others specifically by negative impedance converters with current inversion (INICs), which are indicated in open red arrows. Each node is connected to an external initial signal *V*_*j*(*k*)0_ by a relay *K*_*j*(*k*)_. The two kinds of nodes (green and blue) are grounded in different ways, indicated in green and blue dashed framed rectangles, respectively. (c) The structure of the INIC, consisting of three resistors and an operational amplifier with supplying voltages *V*_+_ and *V*_−_. (d) and (e) Two different ways to ground the nodes, one by a capacitor parallel with an INIC and the other by a capacitor parallel with a normal resistor.

Recently, some investigations [3–7] have shown that the continuous-time quantum walk (CTQW) search algorithm on the graph can also display the quadratic speedup, which is similar to that of Grover's algorithm. Compared with the realizations of Grover's algorithm, CTQW search algorithms are directly connected to the search in a physical database [4] and discrete logic gate operations (e.g., laser pulses) are not necessary. Moreover, spatial search by quantum walk is optimal for almost all graphs [7], which helps to make a flexible design for the search algorithm. An example of the CTQW search algorithm on the complete graph (the “analog analogue” of Grover's algorithm [3, 4]) is shown in Figure 1(a).

We use the *N* vertices of the graph to label computational basis states {|*a*_1_〉, |*a*_2_〉, ⋯, |*a*_*N*_〉} of an *N*-dimensional Hilbert space. The initial state |*s*〉 is an equal superposition of all these basis states as $|s\rangle =1/\sqrt{N}\sum_{n=1}^{N}|a_{n}\rangle$. To find a particular “marked” vertex, or basis state, |*w*〉 given by the oracle Hamiltonian *H*_*w*_ = −|*w*〉〈*w*| (here, we assume that this Hamiltonian is given and we take the first vertex as the marked one for convenience, that is |*w*〉 = |*a*_1_〉 = (1, 0,⋯,0)^T^, the superscript *T* denotes the transposition), the search can be performed by evolving Schrödinger's equation with Hamiltonian *H*_*s*_ = *γL* − |*w*〉〈*w*|, where *γ* is the jumping rate (i.e., amplitude per unit time of the particle transitioning from one vertex to another) and *L* = *D* − *A* is the graph Laplacian. Here, *D* represents the degree matrix (*D*_*jj*_ = deg(*j*) and 0 otherwise), and *A* is the adjacency matrix (*A*_*jk*_ = 1 if *j* and *k* are adjacent, and 0 otherwise). For the regular graphs, *D* is proportional to the unit matrix. We can discard *D* by energy translation without affecting the dynamic behavior of the evolution. Thus, the search Hamiltonian can also be written as [4]:

(1) $$\begin{array}{c} H_{s}=-\gamma A-|w\rangle \langle w|. \end{array}$$

After time *T*, we can get the evolution state |*ψ*(*T*)〉 = *e*^−*iH*_*s*_*T*^|*s*〉. Our objective is to choose the appropriate jumping rate *γ* so that the success probability |〈*w* | *ψ*(*T*)〉|^2^ of finding the target vertex is as close to 1 for as small a time *T* as possible. For the complete graph with *N* vertices, there is a critical jump rate *γ*_*c*_ = 1/*N*, which maximizes the success probability. In such a case, the maximum success probability of finding the target vertex is 1, and the minimum evolution time is $\pi \sqrt{N}/2$ [4, 5].

Up to now, quantum search algorithm has been implemented under many standard quantum circuit models, such as optical experiments [8–13], NMR systems [14, 15], trapped ion [16–18], NV centers [19–21], and superconducting systems [22]. However, these quantum schemes face two bottlenecks: scalability and decoherence. Although there have been some progresses in constructing the number of qubits recently [23, 24], the wide applications within these quantum schemes are still unforeseeable.

On the other hand, recent investigations have shown that classical electric circuits can be used to simulate various topological physics [25–38] and the Schrödinger's equation [39]. It is a conundrum whether the quantum search algorithm can be realized using classical circuits. Since the classical circuit technology is relatively mature, if the quantum search can be realized using electric circuits, it is expected to avoid some problems faced by the quantum schemes.

In this work, we demonstrate that the CTQW search algorithm can be realized by classical electric circuits. The classical circuit networks are designed. As we will show, such search circuit networks have quadratic speedup, which are the same to the quantum schemes. The circuit networks with different sizes are fabricated, and the corresponding searching efficiency is demonstrated experimentally.

## 2. Circuit Theory for Quantum Search

We design a circuit to realize the CTQW search on the complete graph with *N* vertices, as shown in Figure 1(b). The circuit consists of 2*N* nodes denoted by labels *j* = 1, 2, ⋯, *N* and *k* = *N* + 1, *N* + 2, ⋯, 2*N*. Each node is connected to an external initial signal *V*_*j*(*k*)0_ by a relay *K*_*j*(*k*)_. Nodes *j* and *k* for *k* − *j* ≠ *N* as well as for *j* = 1 and *k* = *N* + 1 are connected by negative impedance converters with current inversion (INICs) [29], whose effective resistance is *R*_*jk*_, as specified in Figure 1(c). The detailed description for the function of INICs is given in S1 of Supplementary Materials. The node *j* is grounded by a capacitor *C*_*j*_ parallel with a grounding INIC with effective resistance *R*_*j*0_, while the node *k* is grounded by a capacitor *C*_*k*_ parallel with a normal resistor *R*_*k*0_, as shown in Figures 1(d) and 1(e), respectively. When the relays are turned off, the relations of the currents at the node *j*(*k*) are given by Kirchhoff's current law, as

(2) $$\begin{array}{c} C_{j}\frac{{dV}_{j}}{dt}-\frac{V_{j}}{R_{j0}}=\underset{k}{\sum }\frac{V_{k}-V_{j}}{R_{jk}}, \end{array}$$

(3) $$\begin{array}{c} C_{k}\frac{{dV}_{k}}{dt}+\frac{V_{k}}{R_{k0}}=\underset{j}{\sum }\frac{V_{j}-V_{k}}{-R_{jk}}, \end{array}$$

where *V*_*j*(*k*)_ is the voltage at the node *j* (*k*). By combining the voltages at all nodes, we can define the circuit state as ∣*ϕ*(*t*)) = (*V*_1_(*t*), *V*_2_(*t*),⋯,*V*_2*N*_(*t*))^*T*^. Here, the states in the paper are described by a slightly modified version of the familiar bra-ket notation of quantum mechanics. Then, the set of Eq. (2) and Eq. (3) can be reformulated in the form of a Schrödinger-like equation, as

(4) $$\begin{array}{c} i\partial_{t}\;|\;\phi \left.\left(t\right)\right)=H_{g}\;|\;\phi \left.\left(t\right)\right), \end{array}$$

with *H*_*g*_ being the circuit Hamiltonian. Here, the off-diagonal components of *H*_*g*_ contain the grounding capacitance of node *j* (*k*) and the resistance between nodes *j* and *k*, that is *H*_*g*,*jk*_ = *iC*_*j*_^−1^*R*_*jk*_^−1^ and *H*_*g*,*kj*_ = −*iC*_*k*_^−1^*R*_*jk*_^−1^. The diagonal components are given by the total INICs connected to the node *j* (*k*) as well as the grounding capacitance and INIC (resistance) of node *j* (*k*), as *H*_*g*,*jj*_ = *iC*_*j*_^−1^(*R*_*j*0_^−1^ − ∑_*k*_*R*_*jk*_^−1^) and *H*_*g*,*kk*_ = *iC*_*k*_^−1^(−*R*_*k*0_^−1^ + ∑_*j*_*R*_*jk*_^−1^). As an example, the specific representation of the circuit Hamiltonian *H*_*g*_ of search circuit for the complete graph with 4 vertices is given in S2 of Supplementary Materials.

To match the quantum search Hamiltonian, we now set the parameters of the circuit appropriately. First, all the grounding capacitances are set to *C*_0_ (see S2 of Supplementary Materials). Then, we set the effective resistance of the connecting INIC between nodes *j* and *k* as *R*_*jk*_ = *γ*^−1^*C*_0_^−1^ (*k* − *j* ≠ *N*) and *R*_1,*N*+1_ = *C*_0_^−1^. Here, *γ* is the same to that in Eq. (1). By choosing appropriate grounding INICs (resistances) as *R*_*j*(*k*)0_ = (∑_*k*(*j*)_*R*_*jk*_^−1^)^−1^, the diagonal components of the *H*_*g*_ become zeroes. In such a case, the circuit Hamiltonian can be written as

(5) $$\begin{array}{c} H_{g}=i\left(\begin{array}{cc} O & -H\\ H & O \end{array}\right), \end{array}$$

where *H* is a submatrix with a size of *N* × *N*. With the *N* vertices of the corresponding graph labeling the computational basis states {∣*a*_1_′), |*a*_2_′), ⋯, |*a*_*N*_′)} of the *N*-dimensional graph space, the submatrix *H* can be expressed as

(6) $$\begin{array}{c} H=-\gamma A^{\prime }-\left|w^{\prime }\right)\left(w^{\prime }\right|, \end{array}$$

where *A*′ is the adjacency matrix of the corresponding graph, and |*w*′) = |*a*_1_′) = (1, 0,⋯,0)^T^ represents the target vertex (basis state). Thus, the submatrix *H* in Eq. (6) corresponds to the quantum search Hamiltonian *H*_*s*_ in Eq. (1).

Starting from the initial circuit state ∣*ϕ*(0)) with $V_{j}\left(0\right)=V_{j0}=1/\sqrt{N}$ and *V*_*k*_(0) = *V*_*k*0_ = 0, the evolution circuit state at time *T* can be gotten from Eq. (4) as

(7) $$\begin{array}{c} \left|\phi \left.\left(T\right)\right)\right.=e^{-{iH}_{g}T}\left|\phi \left.\left(0\right)\right)\right.. \end{array}$$

If the jumping rate is set to the critical value *γ*_*c*_ = 1/*N*, we can find the target vertex in a time of $T=\pi \sqrt{N}/2$ with a probability of 1 by projecting the evolution circuit state ∣*ϕ*(*T*)) to the graph space using (1 − *i*) ⊗ *I*_*N*_, where *I*_*N*_ is the identity matrix in the graph space. The detailed demonstration is provided in Methods. The results are consistent with the quantum schemes. However, they are obtained when *R*_*jk*_ and *R*_1,*N*+1_ are fixed as described above. In fact, *R*_*jk*_ and *R*_1,*N*+1_ can take arbitrary values, for example, *R*_*jk*_ = *R*_0_ and *R*_1,*N*+1_ = *γ*_*c*_*R*_0_, here *R*_0_ represents arbitrary resistance. In this case, *H* changes to *H*/(*γC*_0_*R*_0_). If we define an effective time *T*_*e*_ = *T*/(*γC*_0_*R*_0_) instead of the evolution time *T*, the circuit search process is also identical with the standard quantum scheme [3, 4].

**Figure 2:**
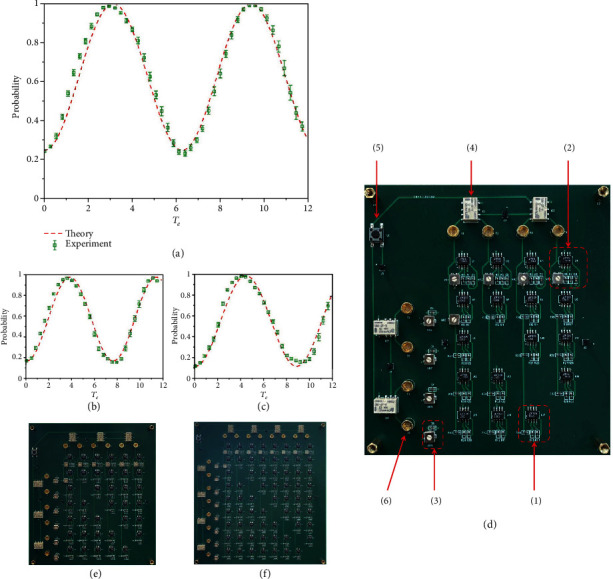
Experimental realization of the search circuits designed for complete graphs. (a) Simulation and experimental results of the circuit search for the complete graph with 4 vertices. The red dashed line represents the theoretical simulation result, while the green square dots represent the experimental data averaged by 20 measurements, with error bars being the variances. (b) and (c) The results of the circuit search for complete graph with 6 and 8 vertices, respectively. (d) A photograph of the printed circuit board (PCB) for the search on complete graph with 4 vertices, with a size of 12∗13 cm^2^. The numbers label some important components. (1) An INIC made of an operational amplifier LT1363 and three surface-mounted device resistors: two *R*_*a*_ = 100 *Ω* and one *R*_*jk*_ = 1 k*Ω*. (2) and (3) Grounding parts for nodes *j* and *k*, respectively. The grounding SMD capacitors are *C*_*j*(*k*)_ = 10 *μ*F. The grounding resistors are replaced by potentiometers. (4) Relays connecting the external initial signals to the nodes. (5) The switch controlling all the relays to initialize the voltages and start the search. (6) Connectors to detect the voltages at all nodes by connecting to an oscilloscope with coaxial cables. (e) PCB for 6 vertices complete graph with a size of 15∗17 cm^2^, (f) PCB for 8 vertices complete graph with a size of 18∗20 cm^2^.

To demonstrate the above theoretical analysis, we perform numerical simulation (with the software LTspice) of the success probabilities of the search on circuits with different sizes. Figures 2(a)–2(c) show the simulated results of success probabilities as a function of effective time *T*_*e*_ for complete graphs with 4, 6, and 8 vertices, respectively. Here, the jumping rate *γ* takes its critical value *γ*_*c*_ = 1/*N* of each model. In the INIC, the operational amplifier LT1363 and two surface-mounted device (SMD) auxiliary resistors with the same value *R*_*a*_ = 100 *Ω* are used. Other parameters of the circuit components are set as *C*_*j*(*k*)_ = 10 *μ*F, *R*_*jk*_ = 1 k*Ω* (except *R*_1,*N*+1_) and *R*_1,*N*+1_ = *γ* k*Ω*. The initial voltages are set as *V*_*j*0_ = 1 V and *V*_*j*0_ = 1 V for convenience. In each figure, the red dashed line represents the evolution of the success probability, where the abscissa is the effective evolution time. As we can see, the shortest effective time we need to complete the search with a success probability close to 1 is approximately 3.1, 3.8, and 4.4 for the three circuits, respectively, which matches the corresponding quantum processes of $\pi \sqrt{N}/2$ for *N* = 4, 6, and 8.

## 3. Experimental Demonstrations

The above theoretical design is easy to realize experimentally. As a proof, we realized the search circuits simulated above on printed circuit boards (PCBs).  Figure 2(d) shows the PCB for the complete graph with 4 vertices in detail, while the corresponding PCBs for complete graphs with 6 and 8 vertices are shown in Figures 2(e) and 2(f). We use the operational amplifier LT1363 and two surface-mounted device (SMD) auxiliary resistors with the same value *R*_*a*_ = 100 *Ω* in the INIC. Other parameters of the circuit components are set as *C*_*j*(*k*)_ = 10 *μ*F and *R*_*jk*_ = 1 k*Ω* (except *R*_1,*N*+1_) in SMD capacitors and resistors. Potentiometers are used instead of normal resistors for grounding resistors *R*_*j*(*k*)0_ and *R*_1,*N*+1_, which are adjusted to the appropriate value. More specific settings of the circuit boards and details of the experiments are given in Methods.

The green square dots in Figures 2(a)–2(c) show the average values of the success probabilities with error bars being the variances. The abscissa represents the effective time. For each circuit, the results are obtained by averaging 20 sets of measurements under the same experimental condition. The average maximum searching success probability for all of the three circuits reaches 0.99 at effective time 3.0, 3.6, and 4.1, respectively. It is seen clearly that the experimental results match with the theoretical simulations well, and they together prove the effectiveness of our design.

**Figure 3:**
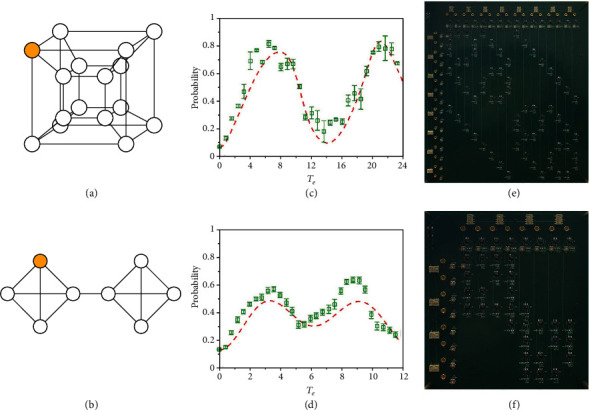
Experimental realization of the search circuits for graphs with other structures. (a) The structure of the 4-dimensional hypercube with 16 vertices. (b) The structure of the joined complete graph with 8 vertices, which is a combination of two 4-vertex complete graphs connected by one edge. (c) Theoretical simulation and experimental data of the circuit search for quantum search on the 4-dimensional hypercube. The jumping rate is taken as 1/4 as an approximation to the critical jumping rate in theory. (d) Theoretical simulation and experimental data of the circuit search for quantum search on the joined complete graph with 8 vertices. The jumping rate is taken as 1/4. (e) PCB for 4-dimensional hypercube with a size of 32∗35 cm^2^. (f) PCB for 8 vertices joined complete graph with a size of 18∗20 cm^2^.

The above discussions only focus on the complete graphs. In fact, such a design is scalable. We can design circuits for quantum search algorithms on various graphs, such as the hypercube [4, 5] and the joined complete graph [6], on which the search algorithms are also optimal [7]. Figures 3(a) and 3(b) show the structures for a 4-dimensional hypercube with 16 vertices and a joined complete graph with 8 vertices, respectively. The corresponding search circuits are designed theoretically and realized experimentally, with the same component models used in the complete graphs. The theoretical simulations and experimental results are given in Figures 3(c) and 3(d), and the PCBs are shown in Figures 3(e) and 3(f). The red dashed lines correspond to the theoretical results, and the green square dots with error bars correspond to the experimental results. Here, the critical jump rate is taken for each structure. The maximum searching success probabilities for the two circuits reach 0.82 and 0.57 at effective time 6.3 and 3.6 (the theoretical results are 0.77 at 7.8 and 0.49 at 3.4, respectively), respectively. As we can see, the experimental results are also in agreement with the theoretical simulations. We have discussed the experimental error in S3 of Supplementary Materials. All the phenomena of our experiments are reproducible. Notice that although more time points (2000 points per turn) were collected by the oscilloscope during the experiments, we have chosen an appropriate density of the points to plot (about 40 points for each circuit), which can not only keep the continuity of the results but also make the results visible and accurate.

## 4. Discussion and Conclusion

In this work, we have implemented the CTQW search algorithm on a classical circuit. The key part of this algorithm is the quantum Schrödinger evolution with Hamiltonian *H*_*s*_, which has been realized in our classical circuit scheme. A full searching process including the “oracle” can be realized by only making some changes to the current scheme. For example, the INIC in the diagonal and the grounding parts should be separate from the main circuit. The position of the INIC in the diagonal determines the search target, which is provided by the “oracle”. The remaining parts form the “driving” circuit corresponding to the “driving” Hamiltonian the quantum scheme has [3]. A more meticulous design and experiment will be implemented in future works.

The number of circuit elements we need in the classical search circuit depends on the connection of the graph structure, i.e., it is proportional to the number of the edges of the graph, which is no more than *O*(*N*^2^) (for complete graph) and can be much smaller in some special cases (e.g., *O*(*N*log*N*) for hypercube). That means, to realize the CTQW algorithm, the resources we need in our classical circuit scheme have the same complexity to that of those quantum schemes as described in Refs. [13, 40].

We would like to point out that although we have chosen the appropriate parameters to make the circuits realizable in our experiments, more variables and their interactions should be considered. For this reason, a multivariate study [41–46] will be down in future works to perform the optimization of the parameters and make the results more stable and more accurate.

In conclusion, we have shown how the CTQW search algorithm can be implemented by using classical electric circuits. The different classical circuit networks to perform the fast search have been designed. The searching efficiencies in the designed classical circuits have been proved to be equivalent to the quantum schemes. Because of the characteristics of the classical circuit, compared with the quantum scheme, the present scheme can show many advantages. For example, it can run in a normal environment without special quantum environments and has good scalability and stability, etc. Moreover, the general design principle in the present work can also be applied, in principle, to other quantum algorithms, even other quantum processes. Thus, the present work is expected to have an important impact on the future information process.

## 5. Methods

### 5.1. The Demonstration of Circuit Search

For the circuit designed for the complete graph with *N* vertices, the search starts from an initial circuit state ∣*ϕ*(0)) = (*V*_1_(0), *V*_2_(0),⋯,*V*_2*N*_(0))^T^, whose components are given by $V_{j}\left(0\right)=1/\sqrt{N}$ (*j* = 1, 2, ⋯, *N*) and *V*_*k*_(0) = 0 (*k* = *N* + 1, *N* + 2, ⋯, 2*N*), and evolves by the circuit Schrödinger-like Eq. (4). ∣*ϕ*(0)) can also be expressed as

(8) $$\begin{array}{c} \left|\phi \left.\left(0\right)\right)\right.=\left(\begin{array}{c} 1\\ 0 \end{array}\right)⊗\left|s^{\prime }\right), \end{array}$$

where $\left|s^{\prime }\right)=1/\sqrt{N}\sum_{n=1}^{N}\left|{a_{n}}^{\prime }\right)$ is an equal superposition of basis states of the graph space, corresponding to the quantum initial state |*s*〉. Thus, from Eq. (7), the circuit state |*ϕ*(*T*)) at time *T* can be calculated as

(9) $$\begin{array}{c} \left|\phi \left.\left(T\right)\right)\right.=e^{-{iH}_{g}T}\left|\phi \left(0\right)\right)=\left(\begin{array}{cc} \cos\left(HT\right) & ‐\sin\left(HT\right)\\ \sin\left(HT\right) & \cos\left(HT\right) \end{array}\right)\left(\begin{array}{c} \left|s^{\prime }\right)\\ O \end{array}\right)=\left(\begin{array}{c} \cos\left(HT\right)\left|s^{\prime }\right)\\ \sin\left(HT\right)\left|s^{\prime }\right) \end{array}\right), \end{array}$$

We then define the circuit search state |*ψ*′(*T*)) by projecting the circuit state to the graph space using $\left(\begin{array}{cc} 1 & -i \end{array}\right)⊗I_{N}$ as

(10) $$\begin{array}{c} \left|\psi^{\prime }\left(T\right)\right)=\left(\begin{array}{cc} 1 & -i \end{array}\right)⊗I_{N}\left|\phi \left(T\right)\right)=\left(\cos\left(HT\right)-i\sin\left(HT\right)\right)\left|s^{\prime }\right)=e^{-iHT}\left|s^{\prime }\right). \end{array}$$

Now we discuss the success probability of finding the target vertex from the circuit search state. Adding −*γI*_*N*_ to the submatrix *H*, which does not affect the observation of the search process, then, we have

(11) $$\begin{array}{c} H=-\gamma N\left|s^{\prime }\right)\left(s^{\prime }\right|-\left|w^{\prime }\right)\left(w^{\prime }\right|. \end{array}$$

If *γ* takes its critical value of *γ*_*c*_ = 1/*N*, then *H* = −|*s*′)(*s*′| − |*w*′)(*w*′|, and its two orthogonal eigenstates are proportional to |*φ*_0_) = |*s*′) + |*w*′) and |*φ*_1_) = |*s*′) − |*w*′) with corresponding eigenvalues $E_{0}=-1-1/\sqrt{N}$ and $E_{1}=-1+1/\sqrt{N}$, respectively.

Since $\left(s^{\prime }\right.\left|w^{\prime }\right)=1/\sqrt{N}$, there are relations $\left(\varphi_{0}|\varphi_{0}\right)=2+2/\sqrt{N}$, $\left(\varphi_{1}|\varphi_{1}\right)=2-2/\sqrt{N}$ and (*φ*_0_ | *φ*_1_) = 0. The inner product of the target state and the circuit search state can be easily calculated as

(12) $$\begin{array}{c} \left(w^{\prime }\;|\;\psi^{\prime }\left(T\right)\right)=\left(\left(\varphi_{0}\;|\;-\left(\varphi_{1}\;|\;\right)\frac{1}{4}\left(e^{-{iE}_{0}T}\;|\;\varphi_{0}\right)+e^{-{iE}_{1}T}\;|\;\varphi_{1}\right)\right)=\frac{1}{2}\left(e^{-{iE}_{0}T}-e^{-{iE}_{1}T}\right)+\frac{1}{2\sqrt{N}}\left(e^{-{iE}_{0}T}+e^{-{iE}_{1}T}\right)=e^{iT}\left(i\sin\left(\frac{T}{\sqrt{N}}\right)+\frac{1}{\sqrt{N}}\cos\left(\frac{T}{\sqrt{N}}\right)\right). \end{array}$$

Thus, the success probability of finding the target state is

(13) $$\begin{array}{c} P\left(T\right)={\left|\left(w^{\prime }\right.\left|\psi^{\prime }\left.\left(T\right)\right)\right.\right|}^{2}=\sin^{2}\left(\frac{T}{\sqrt{N}}\right)+\frac{1}{N}\cos^{2}\left(\frac{T}{\sqrt{N}}\right), \end{array}$$

which also corresponds to the quantum case [3, 4]. It is obvious that the probability reaches 1 at time $T=\pi \sqrt{N}/2$, which is in an order of $O\left(\sqrt{N}\right)$ as the Grover's quantum search algorithm.

### 5.2. Details for Experimental Implementation

We design the PCBs by the software PADS Layout and print it in a local foundry. For the realization of the INIC, we use the high speed, high slew rate operational amplifier (OpAmp) model LT1363 (Linear Technology), whose performance is good enough for our experiments. The OpAmps are supplied by voltages ± 15 V. We use two surface mounted device (SMD) resistors as the auxiliary resistors in the positive and negative feedback loops of the OpAmp. In principle, the values of the two auxiliary resistors can be chosen arbitrarily, provided that they are equal. However, the value should be chosen in a suitable range for the effectiveness of the INIC and the stabilization of the circuit, as the realistic OpAmp has its limited stability conditions. Generally, the auxiliary resistance we take as *R*_*a*_ = 100 *Ω* is appropriate. The values of effective resistors depend on specific situations as we will discuss below.

SMD resistors of 1 k*Ω* are used for the effective resistors of connecting INICs *R*_*jk*_ except *R*_1,*N*+1_. As for the grounding parts, we choose 10 *μ*F SMD capacitors for *C*_0_. We use potentiometers instead of normal resistors for grounding resistors *R*_*j*(*k*)0_ and *R*_1,*N*+1_, due to their unusual required values and some stability requirements (see S3 of Supplementary Materials). The capacitors are produced by Murata company with the 0603 package and are precharacterized to a tolerance of 1% manually. All the resistors are produced by Walsin company with the 0603 package and a tolerance of 1%. The potentiometers are in model 3314J-1 (Bourns) with a range of 0-500 *Ω*.

To initialize the circuit, each node is connected to an external voltage signal *V*_*j*(*k*)0_. We use relay model G6K (Omron) to connect the nodes and the external signals. The relays are controlled by a signal of 12 V through a mechanical switch. With this setting, the external signals can be removed at the same time.

The measured quantities of our circuit are the evolving voltages at all nodes. Thus, we connect the nodes to an oscilloscope by coaxial cables and measure the voltages at all time. We use a 4-channel oscilloscope DSO7104B (Agilent Technologies) in our experiment to collect voltage data. For each circuit, we made three rounds of measurements and at least 20 times per round to verify the reproducibility of the obtained results.

## Data Availability

Any related experimental background information not mentioned in the text and other findings of this study are available from the corresponding author upon reasonable request.

## Supplementary Materials

Supplementary Materials Figure S1: the structure of an INIC, with one operational amplifier and three resistors. Figure S2: the search circuit for the 4 vertices complete graph, with the detail for the currents at the nodes. Figure S3: simulation results for disordered circuits.
