# Supplementary material for: Electric-Circuit Realization of Fast Quantum Search
Source: Research (Wash D C). 2021 Jul 26;2021:9793071. doi: 10.34133/2021/9793071 (PMC8335527; doi:10.34133/2021/9793071)
Supplement: Supplementary Materials — Figure S1: the structure of an INIC, with one operational amplifier and three resistors. Figure S2: the search circuit for the 4 vertices complete graph, with the detail for the currents at the nodes. Figure S3: simulation results for disordered circuits. [file 9793071.f1.docx]

**Supplementary Materials for**

**Electric-circuit realization of fast quantum search**

Naiqiao Pan ^1*^, Tian Chen^1*^, Houjun Sun^2^ and Xiangdong Zhang^1+^

^1^Key Laboratory of Advanced Optoelectronic Quantum Architecture and Measurements of Ministry of Education, Beijing Key Laboratory of Nanophotonics & Ultrafine Optoelectronic Systems, School of Physics, Beijing Institute of Technology, Beijing 100081, China

^2^ Beijing Key Laboratory of Millimeter Wave and Terahertz Techniques, School of Information and Electronics, Beijing Institute of Technology, Beijing 100081, China

*These authors contributed equally to this work. ^+*^Author to whom any correspondence should be

addressed. E-mail: zhangxd@bit.edu.cn; chentian@bit.edu.cn

**S1. Negative impedance converter**

We realize a negative resistance using the negative impedance converter with current inversion (INIC). The INIC between nodes *j* and *k* consists of one operational amplifier (OpAmp) and three resistors, as shown in Fig. S1. The two auxiliary resistors in the positive and negative feedback loops of the OpAmp take the value as *R*_+_ and *R*- respectively, while the third resistor (effective resistor) takes the value as *R_jk_*.


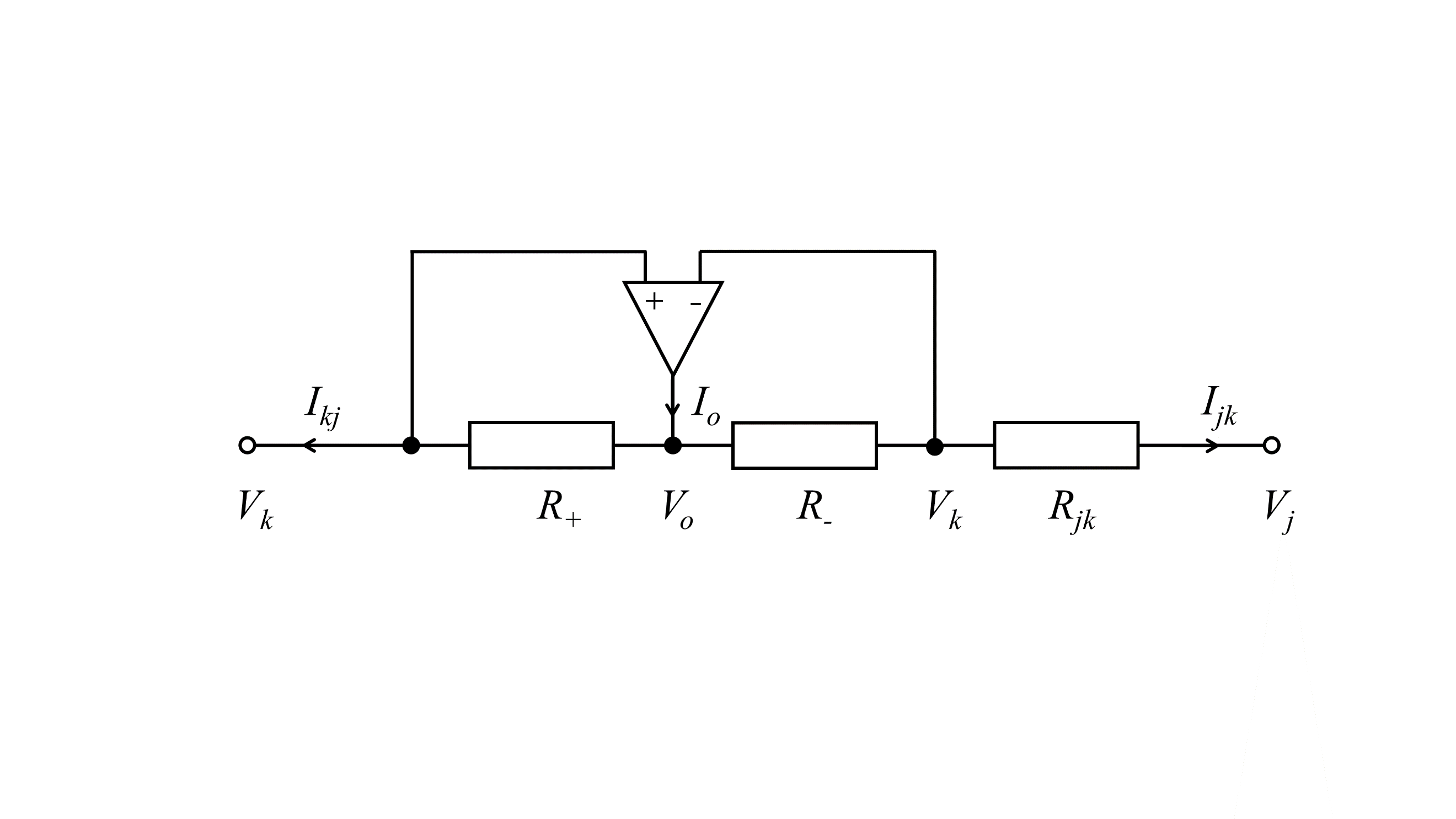


FIGURE S1. The structure of an INIC, with one operational amplifier and three resistors. The directions of the currents and the voltages of the nodes are labeled.

When the OpAmp works in the linear regime, the currents flowing into the inputs of the OpAmp are approximately zero, and the potential at the inverting input equals to that of the non-inverting input, which is the voltage at node *k*. Thus, we can calculate the currents flowing into the nodes $j$ and $k$ as

$I_{jk}=\frac{1}{R_{jk}}\left(V_{k}-V_{j}\right)=\frac{1}{R_{-}}\left(V_{o}-V_{k}\right)$ (S1)

$I_{kj}=\frac{1}{R_{+}}\left(V_{o}-V_{k}\right)$ (S2)

Combining Eqs. (S1) and (S2), we have

$I_{kj}=-\frac{R_{-}}{R_{jk}R_{+}}\left(V_{j}-V_{k}\right)=\frac{R_{-}}{R_{+}}I_{jk}$ (S3)

If we take $R_{+}=R_{-}=R_{a}$, then we have $I_{kj}=I_{jk}$, which means the current at the end near node *k* of the INIC is inverted. Thus, the INIC contributes a negative resistance -*R_jk_* to the current flowing into the node *k*, while it contributes a resistance *R_jk_* to the current flowing into the node *j* as a normal resistor.

As to the grounding INIC connected to the node *j*, the effective resistor of which takes the value as *R_j_*_0_ and it contributes a negative resistance -*R_j_*_0_ to the current flowing away from the node *j*. The use of INICs realizes the anti-symmetry and the zero diagonal elements of the circuit Hamiltonian, which is necessary to our design.

**S2. An example of the circuit design**

In this section we provide the detail for the design of our circuit. We take the search circuit for the complete graph with 4 vertices as an example, which is shown in Fig. S2.


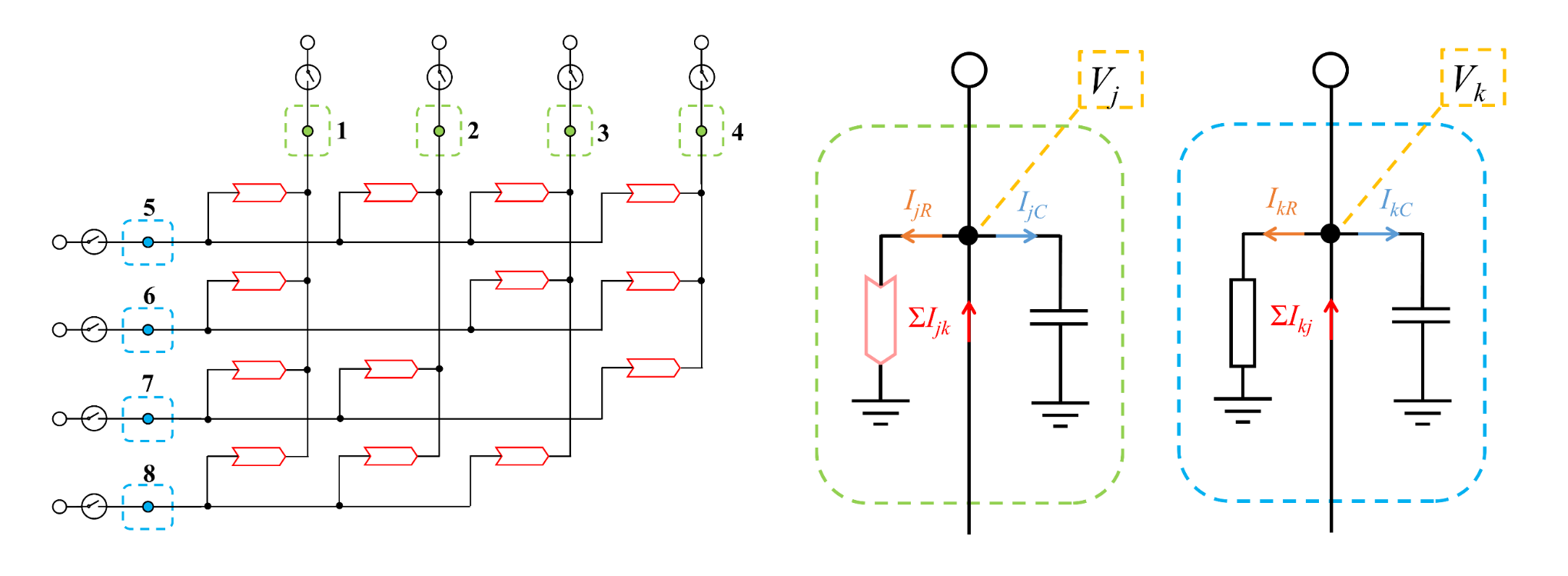


FIGURE S2. The search circuit for the 4 vertices complete graph, with the detail for the currents at the nodes. At each node, the sum of currents in different branches is zero.

From Kirchhoff’s current law, we can write the relations of the currents at all nodes labeling by $j\in\left\{1,2,3,4\right\}$ and $k\in\left\{5,6,7,8\right\}$ as

$I_{j(k)0}=\sum_{k(j)} I_{jk(kj)}$ (S5)

where the left of Eq. (S5) represents the currents flowing away from the node *j* (*k*) and combines two parts as $I_{j(k)0}=I_{j(k)C}+I_{j(k)R}$ with $I_{j(k)C}$ and $I_{j(k)R}$ being the currents flowing through the capacitor and the INIC (or normal resistor), respectively, while the right represents the currents flowing into the node *j* (*k*) from all other nodes connecting to *j* (*k*).

Due to the INICs, the currents can be specifically expressed as:

$I_{j(k)C}=C_{j(k)}\frac{dV_{j(k)}}{dt}$ (S6)

$I_{jR}=\frac{V_{j}}{R_{j0}}$ (S7)

$I_{kR}=-\frac{V_{j}}{R_{j0}}$ (S8)

$I_{jk}=\frac{V_{k}-V_{j}}{R_{jk}}$ (S9)

$I_{kj}=-\frac{V_{j}-V_{k}}{R_{jk}}$ (S10)

Then we can rewrite the current relations into a set of voltage relations as:

$C_{1}\frac{dV_{1}}{dt}=(\frac{1}{R_{1,0}}-\frac{1}{R_{1,5}}-\frac{1}{R_{1,6}}-\frac{1}{R_{1,7}}-\frac{1}{R_{1,8}})V_{1}+\frac{V_{5}}{R_{1,5}}+\frac{V_{6}}{R_{1,6}}+\frac{V_{7}}{R_{1,7}}+\frac{V_{8}}{R_{1,8}}$ (S11)

$C_{2}\frac{dV_{2}}{dt}=(\frac{1}{R_{2,0}}-\frac{1}{R_{2,5}}-\frac{1}{R_{2,7}}-\frac{1}{R_{2,8}})V_{2}+\frac{V_{5}}{R_{2,5}}+\frac{V_{7}}{R_{2,7}}+\frac{V_{8}}{R_{2,8}}$ (S12)

$C_{3}\frac{dV_{3}}{dt}=(\frac{1}{R_{3,0}}-\frac{1}{R_{3,5}}-\frac{1}{R_{3,6}}-\frac{1}{R_{3,8}})V_{3}+\frac{V_{5}}{R_{3,5}}+\frac{V_{6}}{R_{3,6}}+\frac{V_{8}}{R_{3,8}}$ (S13) $C_{4}\frac{dV_{4}}{dt}=(\frac{1}{R_{4,0}}-\frac{1}{R_{4,5}}-\frac{1}{R_{4,6}}-\frac{1}{R_{4,7}})V_{4}+\frac{V_{5}}{R_{4,5}}+\frac{V_{6}}{R_{4,6}}+\frac{V_{7}}{R_{4,7}}$ (S14)

$C_{5}\frac{dV_{5}}{dt}=-\frac{V_{1}}{R_{1,5}}-\frac{V_{2}}{R_{2,5}}-\frac{V_{3}}{R_{3,5}}-\frac{V_{4}}{R_{4,5}}\text{+}(-\frac{1}{R_{5,0}}+\frac{1}{R_{1,5}}+\frac{1}{R_{2,5}}+\frac{1}{R_{3,5}}+\frac{1}{R_{4,5}})V_{5}$ (S15) $C_{6}\frac{dV_{6}}{dt}=-\frac{V_{1}}{R_{1,6}}-\frac{V_{3}}{R_{3,6}}-\frac{V_{4}}{R_{4,6}}+(-\frac{1}{R_{6,0}}+\frac{1}{R_{1,6}}+\frac{1}{R_{3,6}}+\frac{1}{R_{4,6}})V_{6}$ (S16)

$C_{7}\frac{dV_{7}}{dt}=-\frac{V_{1}}{R_{1,7}}-\frac{V_{2}}{R_{2,7}}-\frac{V_{4}}{R_{4,7}}+(-\frac{1}{R_{7,0}}+\frac{1}{R_{1,7}}+\frac{1}{R_{2,7}}+\frac{1}{R_{4,7}})V_{7}$ (S17)

$C_{8}\frac{dV_{8}}{dt}=-\frac{V_{1}}{R_{1,8}}-\frac{V_{2}}{R_{2,8}}-\frac{V_{3}}{R_{3,8}}+(-\frac{1}{R_{8,0}}+\frac{1}{R_{1,8}}+\frac{1}{R_{2,8}}+\frac{1}{R_{3,8}})V_{8}$ (S18)

The voltage relations constitute a system of the first-order linear differential equations, which can be reformulated in the form of the Schrödinger-like equation with and the circuit Hamiltonian

$H_{g}=i\left(\begin{matrix} Y_{1} & 0 & 0 & 0 & \frac{1}{C_{1}R_{1,5}} & \frac{1}{C_{1}R_{1,6}} & \frac{1}{C_{1}R_{1,7}} & \frac{1}{C_{1}R_{1,8}} \\ 0 & Y_{2} & 0 & 0 & \frac{1}{C_{2}R_{2,5}} & 0 & \frac{1}{C_{2}R_{2,7}} & \frac{1}{C_{2}R_{2,8}} \\ 0 & 0 & Y_{3} & 0 & \frac{1}{C_{3}R_{3,5}} & \frac{1}{C_{3}R_{3,6}} & 0 & \frac{1}{C_{3}R_{3,8}} \\ 0 & 0 & 0 & Y_{4} & \frac{1}{C_{4}R_{4,5}} & \frac{1}{C_{4}R_{4,6}} & \frac{1}{C_{4}R_{4,7}} & 0 \\ -\frac{1}{C_{5}R_{1,5}} & -\frac{1}{C_{5}R_{2,5}} & -\frac{1}{C_{5}R_{3,5}} & -\frac{1}{C_{5}R_{4,5}} & Y_{5} & 0 & 0 & 0 \\ -\frac{1}{C_{6}R_{1,6}} & 0 & -\frac{1}{C_{6}R_{3,6}} & -\frac{1}{C_{6}R_{4,6}} & 0 & Y_{6} & 0 & 0 \\ -\frac{1}{C_{7}R_{1,7}} & -\frac{1}{C_{7}R_{2,7}} & 0 & -\frac{1}{C_{7}R_{4,7}} & 0 & 0 & Y_{7} & 0 \\ -\frac{1}{C_{8}R_{1,8}} & -\frac{1}{C_{8}R_{2,8}} & -\frac{1}{C_{8}R_{3,8}} & 0 & 0 & 0 & 0 & Y_{8} \end{matrix}\right)$ (S19)

where $Y_{1}=-\frac{1}{R_{1,0}}-\frac{1}{R_{1,5}}-\frac{1}{R_{1,6}}-\frac{1}{R_{1,7}}-\frac{1}{R_{1,8}}$ and so on.

To form the circuit Hamiltonian we need the opposite of the [upper right](http://www.baidu.com/link?url=l_Fqjv7ulpKq2c27g7NW5UtUeabZ7_R3_CQ0Y8wOY-uL_SISFxlrXVdeNulqb1mU8zcloFAyBKvxPlpEXmZJXpgoiGzKh1XwocMBZL9EZKO) submatrix in Eq. (5) of the main text.

$-H_{u}\text{=}\left(\begin{matrix} -\frac{1}{C_{1}R_{1,5}} & -\frac{1}{C_{1}R_{1,6}} & -\frac{1}{C_{1}R_{1,7}} & -\frac{1}{C_{1}R_{1,8}} \\ -\frac{1}{C_{2}R_{2,5}} & 0 & -\frac{1}{C_{2}R_{2,7}} & -\frac{1}{C_{2}R_{2,8}} \\ -\frac{1}{C_{3}R_{3,5}} & -\frac{1}{C_{3}R_{3,6}} & 0 & -\frac{1}{C_{3}R_{3,8}} \\ -\frac{1}{C_{4}R_{4,5}} & -\frac{1}{C_{4}R_{4,6}} & -\frac{1}{C_{4}R_{4,7}} & 0 \end{matrix}\right)$ (S20)

should correspond to the search Hamiltonian

$H\text{=}\gamma\left(\begin{matrix} -\frac{1}{\gamma} & -1 & -1 & -1 \\ -1 & 0 & -1 & -1 \\ -1 & -1 & 0 & -1 \\ -1 & -1 & -1 & 0 \end{matrix}\right)$ (S21)

which means the corresponding elements of the two matrices are equal. Then we have

$\frac{1}{C_{1}R_{1,6}}=\frac{1}{C_{1}R_{1,7}}=\frac{1}{C_{1}R_{1,8}}=\gamma$ (S22)

Meanwhile, the lower left submatrix of the circuit Hamiltonian

$H_{l}\text{=}\left(\begin{matrix} -\frac{1}{C_{5}R_{1,5}} & -\frac{1}{C_{5}R_{2,5}} & -\frac{1}{C_{5}R_{3,5}} & -\frac{1}{C_{5}R_{4,5}} \\ -\frac{1}{C_{6}R_{1,6}} & 0 & -\frac{1}{C_{6}R_{3,6}} & -\frac{1}{C_{6}R_{4,6}} \\ -\frac{1}{C_{7}R_{1,7}} & -\frac{1}{C_{7}R_{2,7}} & 0 & -\frac{1}{C_{7}R_{4,7}} \\ -\frac{1}{C_{8}R_{1,8}} & -\frac{1}{C_{8}R_{2,8}} & -\frac{1}{C_{8}R_{3,8}} & 0 \end{matrix}\right)$ (S23)

should also correspond to $H_{s}$, which makes

$\frac{1}{C_{6}R_{1,6}}=\frac{1}{C_{7}R_{1,7}}=\frac{1}{C_{8}R_{1,8}}=\gamma$ (S24)

Comparing Eq. (S22) with Eq. (S24), we have

$R_{1,6}=R_{1,7}=R_{1,8}=\frac{1}{\gamma C_{1}}$ (S25)

and

$C_{6}=C_{7}=C_{8}=\frac{1}{\gamma R_{1,6}}=C_{1}$ (S26)

With some similar derivations, we can get the conclusion that all the capacitances should be equal, which we set to $C_{0}$, and all the effective resistances of the connecting INICs (except $R_{1,5}$) should be equal too, which we set to $\frac{1}{\gamma C_{0}}$. Additionally, we set $R_{1,5}=\frac{1}{C_{0}}$ and the grounding resistances

$R_{1,0}=R_{5,0}=\frac{1}{(1+3\gamma)C_{0}}$ (S27)

$R_{2,0}=R_{3,0}=R_{4,0}=R_{6,0}=R_{7,0}=R_{8,0}=\frac{1}{3\gamma C_{0}}$ (S28)

With the above settings, we have

$H_{g}=i\left(\begin{matrix} 0 & -H \\ H & 0 \end{matrix}\right)$ (S29)

where *H* corresponds to *H_s_*.

The same method can be applied to bigger complete graphs and other structures, e.g., the 4-dimensional hypercube (16 vertices) with the search Hamiltonian

$H_{s}'=-\gamma\left(\begin{matrix} 1/\gamma& 1 & 1 & 0 & 1 & 0 & 0 & 0 & 1 & 0 & 0 & 0 & 0 & 0 & 0 & 0 \\ 1 & 0 & 0 & 1 & 0 & 1 & 0 & 0 & 0 & 1 & 0 & 0 & 0 & 0 & 0 & 0 \\ 1 & 0 & 0 & 1 & 0 & 0 & 1 & 0 & 0 & 0 & 1 & 0 & 0 & 0 & 0 & 0 \\ 0 & 1 & 1 & 0 & 0 & 0 & 0 & 1 & 0 & 0 & 0 & 1 & 0 & 0 & 0 & 0 \\ 1 & 0 & 0 & 0 & 0 & 1 & 1 & 0 & 0 & 0 & 0 & 0 & 1 & 0 & 0 & 0 \\ 0 & 1 & 0 & 0 & 1 & 0 & 0 & 1 & 0 & 0 & 0 & 0 & 0 & 1 & 0 & 0 \\ 0 & 0 & 1 & 0 & 1 & 0 & 0 & 1 & 0 & 0 & 0 & 0 & 0 & 0 & 1 & 0 \\ 0 & 0 & 0 & 1 & 0 & 1 & 1 & 0 & 0 & 0 & 0 & 0 & 0 & 0 & 0 & 1 \\ 1 & 0 & 0 & 0 & 0 & 0 & 0 & 0 & 0 & 1 & 1 & 0 & 1 & 0 & 0 & 0 \\ 0 & 1 & 0 & 0 & 0 & 0 & 0 & 0 & 1 & 0 & 0 & 1 & 0 & 1 & 0 & 0 \\ 0 & 0 & 1 & 0 & 0 & 0 & 0 & 0 & 1 & 0 & 0 & 1 & 0 & 0 & 1 & 0 \\ 0 & 0 & 0 & 1 & 0 & 0 & 0 & 0 & 0 & 1 & 1 & 0 & 0 & 0 & 0 & 1 \\ 0 & 0 & 0 & 0 & 1 & 0 & 0 & 0 & 1 & 0 & 0 & 0 & 0 & 1 & 1 & 0 \\ 0 & 0 & 0 & 0 & 0 & 1 & 0 & 0 & 0 & 1 & 0 & 0 & 1 & 0 & 0 & 1 \\ 0 & 0 & 0 & 0 & 0 & 0 & 1 & 0 & 0 & 0 & 1 & 0 & 1 & 0 & 0 & 1 \\ 0 & 0 & 0 & 0 & 0 & 0 & 0 & 1 & 0 & 0 & 0 & 1 & 0 & 1 & 1 & 0 \end{matrix}\right)$ (S30)

and the 8 vertices joined the complete graph with the search Hamiltonian

$H_{s}''=-\gamma\left(\begin{matrix} 1/\gamma& 1 & 1 & 1 & 0 & 0 & 0 & 0 \\ 1 & 0 & 1 & 1 & 0 & 0 & 0 & 0 \\ 1 & 1 & 0 & 1 & 0 & 0 & 0 & 0 \\ 1 & 1 & 1 & 0 & 1 & 0 & 0 & 0 \\ 0 & 0 & 0 & 1 & 0 & 1 & 1 & 1 \\ 0 & 0 & 0 & 0 & 1 & 0 & 1 & 1 \\ 0 & 0 & 0 & 0 & 1 & 1 & 0 & 1 \\ 0 & 0 & 0 & 0 & 1 & 1 & 1 & 0 \end{matrix}\right)$ (S31)

Here, the joined complete graph is approximated as a regular graph for convenience, so the diagonal components of the search Hamiltonian have only one non-zero element.

**S3. Explanation for the** **experimental error**

Although our theoretical derivation is perfect, there are still experimental errors compared with the theory. Here we study and explain some main reasons for the experimental errors, and show the measures we take to reduce the errors.

1. *Disorder of the circuit components*

The components we use in the experiments, including the capacitors and the resistors, are disordered by nature due to the limitation of the production technology. Generally speaking, the disorder of the components leads to a disorder of the circuit Hamiltonian, which further affects the dynamic behavior of circuit evolution. We study the influence of disorder on the search success probability of the circuits by simulation, the results of which is shown in Fig. S3. In the simulations, the disorder of the circuits is characterized by the tolerance of the components used in the circuits. The capacitors and the resistors are added with varying degrees of tolerance, from 0 to 10%. The simulation results show that under the influence of disorder, the success probability and search time of the search circuit deviate from the theoretical value and the deviation increases with the disorder degree and evolution time. The scale and the graph structure also affect the error caused by disorder.

In our experiments, we choose the capacitors and the resistors with tolerance of 1%, which greatly reduces the error caused by disorder.

The disorder also comes from the adjustment accuracy of the potentiometers we use. The adjustment of the potentiometers is not arbitrary, which leads to a tolerance of 5% on average by measurement.


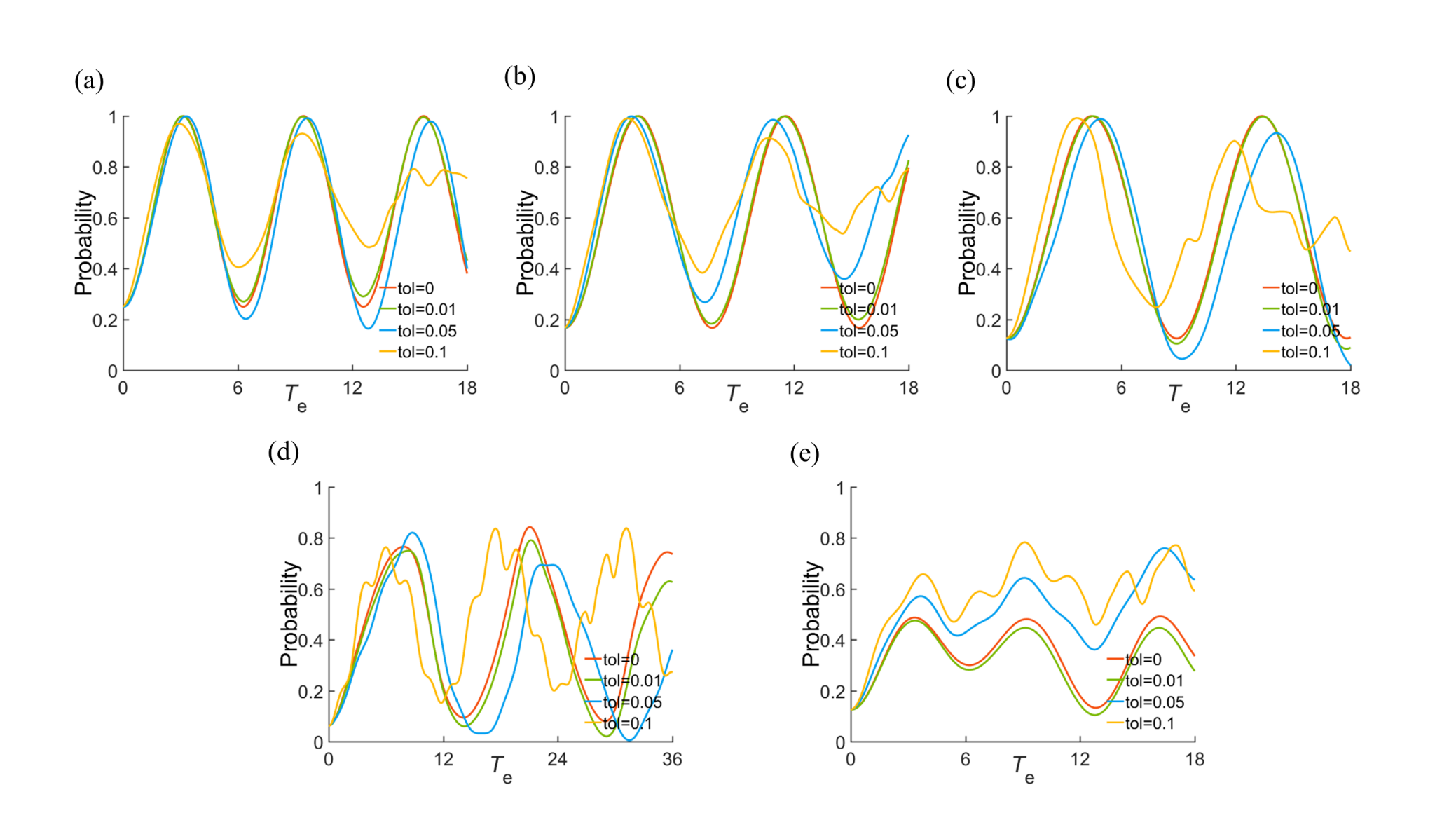


FIGURE S3. Simulation results for disordered circuits. (a), (b), (c), (d) and (e) are the simulation results of search success probability under different degrees of disorder for complete graph with 4, 6, 8 vertices, 4-dimensional hypercube and joined complete graph with 8 vertices, respectively. The red, green, blue and yellow solid line show the results of components (capacitors and resistors) with tolerance 0 (ideal), 1%, 5% and 10%, respectively. The legend ‘tol’ means tolerance.

1. *Parasitic effect*

Parasitic effect, especially the parasitic resistances (as our circuits work under DC-like conditions) which were ignored in the theoretical deductions and simulations, will also affect the evolution of the circuits. The parasitic resistance of a capacitor refers to the resistance formed by its leads and plates, which is inevitable for an actual capacitor. Meanwhile, the leads inside the PCBs also create additional resistances.

The most important influence of the parasitic resistances is that they create non-zero diagonal elements of the circuit Hamiltonian, which makes the Hamiltonian non-Hermitian and further leads to the decay of the voltage signals in the circuit evolution.

As a countermeasure, we replace the resistors (and INIC effective resistors) with potentiometers in the grounding parts. By adjusting the potentiometers manually, we can re-zero the diagonal elements of the circuit Hamiltonian and make the voltage signals evolve for a longer time.

1. *Delay of the signals*

For an actual circuit, the transmission of the signals may be delayed by some circuit elements, e.g., the OpAmps and the relays. To reduce the impact of such instabilities, we choose circuit elements with short delay. The propagation delay of the OpAmp model LT1363 is less than 5 ns, far less than the evolution time. The release time of the relay model G6K is approximately 1.3 ms, while the difference of the release time for different relays is less than 0.1 ms by measurement. Since the delay time is basically unchanged, we can extend the evolution time by choosing larger capacitances and resistances to reduce the impact of delay. We choose 10 μF for the capacitances and 1 kΩ for the connecting INIC resistances (except *R*_1_,*_N_*_+1_), with which the evolution time is in an order of 10 ms, much larger than the delay time of the elements.
